# The immune landscape of melanoma microenvironmental crosstalk

**DOI:** 10.3389/fimmu.2026.1847963

**Published:** 2026-05-11

**Authors:** Qianqian Yang, Qingjing Yang, Bolin Xu, Xingyu Chen, Huilan Zheng, Mingming Wang, Winjin Zhang, Jingping Wu, Hongbin Cheng

**Affiliations:** 1 Clinical Medical College, Chengdu University of Traditional Chinese Medicine, Chengdu, Sichuan, China; 2 Department of Medical Aesthetics, Affiliated Hospital of Chengdu University of Traditional Chinese Medicine, Chengdu, Sichuan, China; 3 Department of Acupuncture and Moxibustion, Zhaoyang District Hospital of Traditional Chinese Medicine, Zhaotong, Yunnan, China; 4 Department of Dermatology, Hospital of Chengdu University of Traditional Chinese Medicine, Chengdu University of Traditional Chinese Medicine, Chengdu, Sichuan, China

**Keywords:** cancer-associated fibroblasts, extracellular matrix, immune cells, immunotherapy, melanoma, tumor microenvironment, tumor-associated macrophages

## Abstract

Melanoma is a highly aggressive skin malignancy characterized by early metastasis, marked therapeutic resistance, and poor clinical outcomes in advanced disease. Increasing evidence indicates that immune dysregulation within the tumor microenvironment (TME) is a central determinant of melanoma progression, metastatic dissemination, and treatment failure. The melanoma immune microenvironment is shaped by complex interactions among tumor-associated macrophages, tumor-infiltrating lymphocytes, regulatory T cells, myeloid-derived suppressor cells, stromal cells, endothelial cells, and extracellular matrix components, together with hypoxia and acidosis, all of which cooperatively drive immune evasion, chronic inflammation, angiogenesis, and resistance to therapy. Mechanistically, cancer-associated fibroblasts promote immune tolerance by secreting CXCL12 and CCL17, thereby recruiting regulatory T cells and reinforcing a suppressive stromal niche; melanoma-derived factors and metabolic perturbations reprogram tumor-associated macrophages toward a pro-tumoral phenotype through lipid metabolic remodeling, endoplasmic reticulum stress, and immunosuppressive mediator production; and hypoxic stress amplifies CD39/CD73-dependent adenosine generation, which suppresses dendritic cell maturation and cytotoxic T-cell activity while facilitating immune escape. These immunosuppressive networks not only impair effective antitumor immune surveillance but also limit the long-term efficacy of immune checkpoint blockade and other systemic treatments. This review summarizes the immunopathological mechanisms underlying melanoma progression within the immune microenvironment and highlights emerging therapeutic strategies targeting immune and stromal crosstalk, providing a valuable theoretical basis for understanding melanoma immune escape and an important reference for the development of more effective and durable immunotherapeutic interventions.

## 1 Introduction

Melanoma is one of the most aggressive forms of skin cancer, distinguished by its strong metastatic potential, high degree of biological heterogeneity, and frequent therapeutic resistance, particularly in advanced-stage disease (1). Although substantial progress has been achieved with targeted therapies and immune checkpoint inhibitors, durable clinical responses remain limited to a subset of patients, and treatment failure caused by primary or acquired resistance continues to represent a major challenge (2). These limitations underscore the need to move beyond a tumor cell–centered perspective and to better understand melanoma as a complex ecosystem shaped by continuous bidirectional interactions between malignant cells and their surrounding microenvironment (3). Among the diverse components of the melanoma tumor microenvironment, the immune microenvironment has emerged as a central regulator of tumor initiation, progression, immune escape, metastatic dissemination, and therapeutic responsiveness (4, 5).

Tumor-associated macrophages, regulatory T cells, myeloid-derived suppressor cells, dysfunctional cytotoxic lymphocytes, stromal fibroblasts, endothelial cells, extracellular matrix remodeling, hypoxia, and acidosis collectively establish an immunosuppressive niche that restrains effective antitumor immunity while supporting melanoma cell survival and invasion (6, 7). Increasing evidence indicates that these cellular and molecular networks are not merely secondary consequences of tumor growth, but active drivers of disease behavior and key determinants of immunotherapy efficacy (8). This review focuses on the immune microenvironment of the melanoma microenvironment, with particular emphasis on the reciprocal crosstalk between immune and stromal components.

## 2 Roles of the extracellular matrix in melanoma initiation and progression

Macromolecules discharged by cells populate the extracellular void, collectively assembling into a dense, linked web known as the ECM. Broad categorization divides this structure into the stromal framework and the basement layer, where collagens serve as the main architectural elements. Within malignant melanoma contexts, equilibrium of the matrix differs significantly from healthy dermal tissue, often disrupted by enzymatic breakdown processes involving matrix metalloproteinases (MMPs). Heightened collagen concentration alongside augmented tissue rigidity facilitates neoplastic cell expansion and metastatic spread, consequently diminishing overall prognosis (9). Basement membranes primarily contain type IV collagen as a major building block. Stromal populations within tumors show elevated levels of COL4A1, linking directly to higher regulatory T cell and M2 macrophage presence, plus augmented release of suppressive signaling molecules (10). Conversely, within the connective tissue framework, upregulated type I collagen levels link to aggressive tumor characteristics (11). Importantly, various melanoma variants display different behaviors regarding mechanical properties and basement-layer adjustments. As malignancy evolves, swift alterations in basement layers accompany sustained low tissue rigidity. Such physical conditions absorb stress and harm caused by fast expanding lesions, potentially explaining why spread to distant sites often stays contained (12). Conversely, advancing disease shows diminished basal reconstruction and creation, resulting in a firmer underlying matrix; likewise, excess keratin levels specifically fortify the stratum corneum. This inflexible, layered configuration proves structurally unsound throughout tumor development, favoring membrane breakage and enzymatic destruction of the matrix, ultimately enhancing metastatic potential (12).

## 3 Stromal cell populations in the melanoma landscape

### 3.1 The pivotal role of cancer-associated fibroblasts

**Figure 1:**
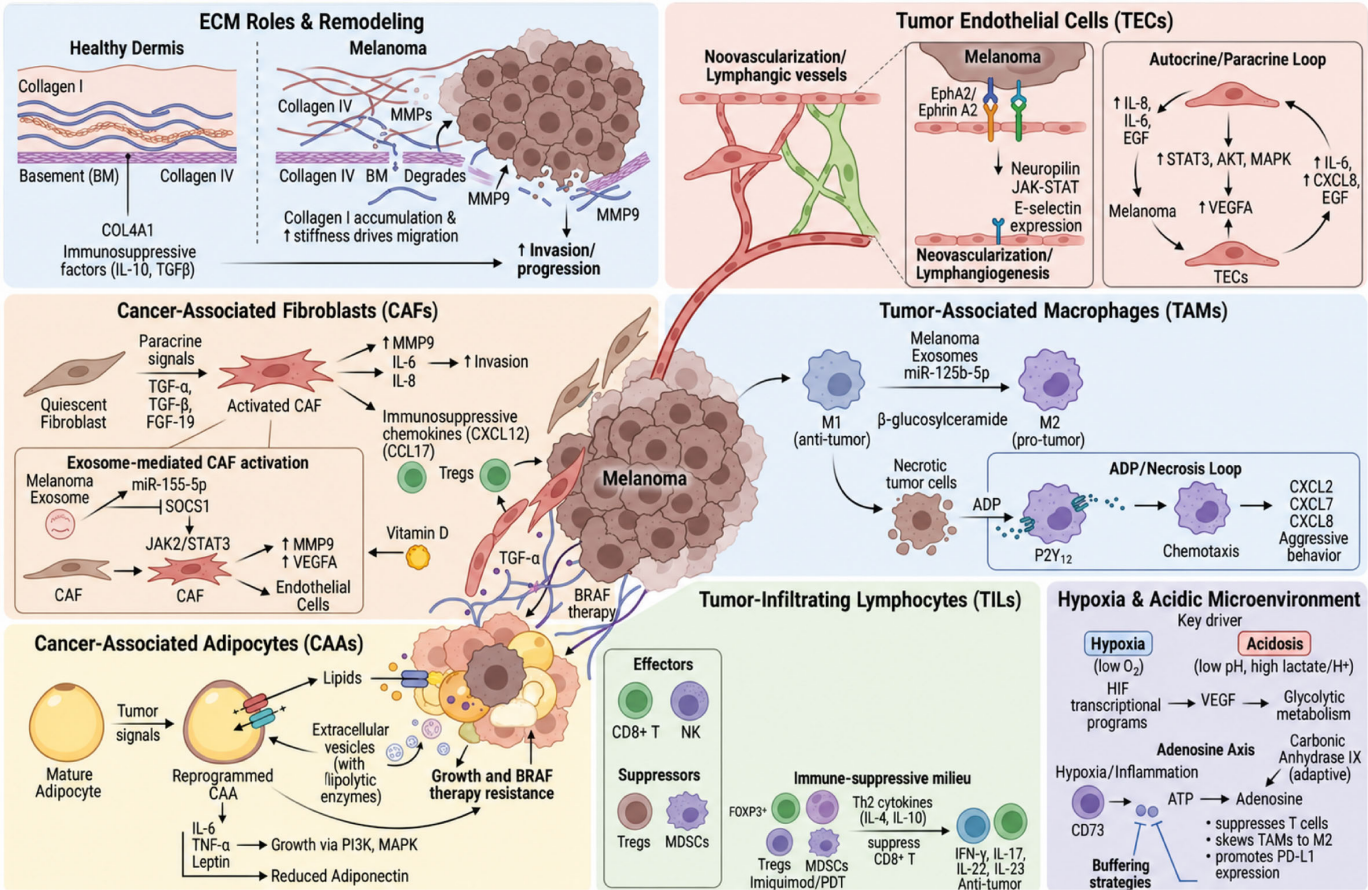
The immune landscape of melanoma microenvironmental crosstalk.

Cancer-associated fibroblasts (CAFs) constitute the predominant mesenchymal cellular cohort residing within the tumor microenvironment. Extensive proteomic profiling of CAF-related markers across various melanoma subtypes reveals significant heterogeneity in their phenotypic profiles. For instance, while platelet-derived growth factor receptor β (PDGFRβ) expression remains minimal in certain melanoma contexts, it is markedly upregulated in others (13). A bidirectional regulatory crosstalk exists between malignant cells and CAFs. Neoplastic cells facilitate the paracrine release of TGF-α, TGF-β, and fibroblast growth factor 19 (FGF-19), signaling cascades that drive the differentiation of quiescent fibroblasts into activated CAFs (14). Conversely, these activated stromal cells accelerate melanoma advancement through extracellular matrix (ECM) reorganization and the secretion of pro-inflammatory cytokines, including IL-1α, IL-1β, IL-6, and IL-8 (15). Crucially, three-dimensional spheroid invasion models demonstrated that concurrent inhibition of both IL-6 and IL-8 effectively eliminates CAF-driven invasive capabilities in human melanoma lines (16). Furthermore, exosomes derived from melanoma cells carrying miR-155-5p suppress the expression of suppressor of cytokine signaling 1 (SOCS1). This suppression triggers the JAK2/signal transducer and activator of STAT3 signaling axis, leading to elevated levels of vascular endothelial growth factor A (VEGFA) and MMP9 within CAFs; this cascade subsequently fuels endothelial cell migration, proliferation, and neovascularization (17). In both melanoma lesions and surrounding non-neoplastic skin, CAFs exhibit heightened production of immunosuppressive chemokines, specifically CXCL12 and CCL17. These factors attract regulatory T cells (Tregs), thereby fostering an immune-tolerant niche conducive to tumor expansion (18). Additionally, vitamin D serves as a critical modulator of cutaneous homeostasis and has been identified as a potential suppressor of CAF activation; consequently, deficient vitamin D status correlates with an elevated susceptibility to melanoma development (8, 19) (**Figure 1**).

### 3.2 The influence of cancer-associated adipocytes on melanoma

Cancer-associated adipocytes (CAAs) arise when peri-invasive adipose tissue is reprogrammed by tumor-derived paracrine signals, thereby establishing a microenvironment that supports melanoma progression (20). These altered adipocytes promote tumor growth by reshaping the local metabolic and immune milieu through the release of lipids, bioactive mediators, adipokines, and other soluble factors. In co-culture systems, melanoma cells exhibit increased intracellular lipid accumulation, indicating that adipocyte-derived lipid transfer confers a survival and proliferative advantage. Mechanistically, lipid uptake is mediated by membrane transporters and activates PTEN-dependent Akt signaling to facilitate tumor expansion (21). In parallel, melanoma cells internalize adipocyte-derived extracellular vesicles containing lipolytic enzymes, further enhancing aggressive behavior (22). Aberrant lipid metabolism is also closely linked to therapeutic resistance. Enzymes involved in lipid synthesis are upregulated during metastatic progression, whereas their depletion restores sensitivity to BRAF-targeted therapy (23). Beyond metabolic support, CAAs secrete pro-inflammatory cytokines, including IL-6 and TNF-α, which activate oncogenic signaling pathways that accelerate tumor development (24). Leptin further promotes melanoma cell proliferation and resistance through PI3K- and MAPK-dependent signaling, and elevated leptin receptor expression correlates with greater disease severity (25–27). By contrast, adiponectin exerts tumor-suppressive effects, yet both adiponectin and its receptors are reduced in cutaneous malignancies (28). Moreover, stromal adipose and fibrotic infiltration can impede drug delivery, as observed in vismodegib-resistant tumors (29). Adipose tissue also activates Wnt-5a signaling and undergoes fibroblast-like transdifferentiation, thereby enhancing melanoma cell motility (30).

### 3.3 Tumor endothelial cells in melanoma

Specialized endothelial components derived from tumors assume a fundamental function within neovascular processes and lymphatic channel development, supplying essential oxygen resources alongside metabolic substrates to malignant entities while enabling distant dissemination. Pathological evolution encompasses multiple steps wherein cancerous cellular units penetrate blood vessels via intravasation, traverse systemic transport systems, and form secondary lesions following extravasation. In melanocytic malignancies, triggering signals through alternative ephrin A2 receptors strengthens interaction capacity between transformed cells and vessel walls while simultaneously elevating vascular wall permeability characteristics, which supports transcellular crossing (31). Furthermore, reduced representation of neuropilin-associated proteins within both endothelial compartments and smooth muscle populations triggers activation of the JAK-STAT cascade, specifically driving selective gene expression for E-selectin on vascular surfaces and reinforcing adhesive interactions between malignant and stromal elements (32). The plasminogen activation apparatus, notably present in cutaneous cancers, contributes to new vessel formation by amplifying mitogenic signaling events involving ERK1/2 kinases within endothelial populations (33, 34). Endothelial-derived tumor components additionally support immune system bypass mechanisms through elevated display of major histocompatibility complex-related antigen presentation sequences, resulting in elimination of cytotoxic CD8+ lymphocytes and inhibition of regulatory T-cell populations (35). Beyond these direct effects, soluble factors released from TECs including interleukin-8, IL-6, and epidermal growth factor activate intracellular signaling networks containing STAT3, AKT, and MAPK pathways within tumor cells, driving enhanced VEGF production. This heightened growth factor subsequently stimulates further secretion of IL-6, CXCL8, and EGF from vascular elements themselves, establishing a self-reinforcing molecular circuit favoring widespread dissemination throughout tissues while preventing detachment-induced cell death (36).

### 3.4 Melanoma interactions with tumor-associated macrophages

Within the complex landscape of tumor-infiltrating immune elements, macrophage subtypes stand out as the predominant cellular cohort inhabiting the tumor niche. These cells are broadly classified into two functional phenotypes: one exhibiting anti-neoplastic properties aligned with M1 markers and another fostering malignant progression similar to M2 profiles. Early-stage tumors typically harbor M1-dominant populations, while later disease stages demonstrate a shift toward M2 predominance (37). Conditional deletion of Patched1 in murine models revealed that M1-like macrophages cooperate with dendritic cells to suppress tumoral expansion; their depletion correlates with accelerated disease progression. Additionally, melanoma-secreted exosomes carrying miR-125b-5p target lysosomal acid lipase A expression in macrophages, effectively reprogramming them toward an M2-skewed phenotype (38). The presence of β-glucosylceramide derived from melanoma cells further drives M2 polarization through enhanced sterol synthesis, disruption of endoplasmic reticulum membrane lipid composition, and induction of ER stress responses (39). Functionally, these macrophage subsets impede host antitumor defenses by secreting indoleamine 2,3-dioxygenase, which depletes tryptophan pools, restricts T-cell clonal expansion, and facilitates regulatory T-cell recruitment within the tumor bed (40). Hypoxic zones within melanomas accumulate nucleotides released during necrosis of both malignant and stromal cells. Adenosine diphosphate functions as a chemotactic “find-me” signal, intensifying migration of P2Y12-expressing macrophages toward necrotic regions. Upon ADP detection, these activated macrophages release pro-inflammatory CXCL2, CXCL7, and CXCL8 cytokines that collectively stimulate aggressive tumor behavior (41). Furthermore, TNF-α produced by macrophages acts as a critical survival factor for melanoma cells while contributing to therapeutic resistance against MAPK pathway inhibitors (42). Beyond cytokine signaling, MMP1 and MMP2 proteases alongside soluble factors such as IL-1, EGF, PDGF, and VEGF orchestrate angiogenic sprouting and invasive capabilities.

### 3.5 Tumor-infiltrating lymphocytes melanoma

Tumor-infiltrating lymphoid populations in melanoma comprise functionally distinct immune subsets that either support or restrain antitumor immunity (43, 44). Effector populations, including CD4^+^ T cells, CD8^+^ T cells, and NK cells, contribute to tumor control, whereas Tregs and myeloid-derived suppressor cells (MDSCs) suppress host immune surveillance (44). High-dimensional imaging using cyclic microscopy and real-time virtual histology has further revealed that the spatial organization of these cells is not random; rather, keratinocyte-mediated penetration and intercellular junctional anchoring can sequester superficial CD8^+^ T cells beneath the epidermal barrier, thereby limiting their cytotoxic access to melanoma cells and facilitating immune escape (45, 46). Clinically, increased infiltration of CD4^+^ T cells, CD8^+^ T cells, and CD20^+^ B cells is associated with improved prognosis, consistent with the role of CD4^+^ helper T cells in sustaining CD8^+^ cytotoxic activation (44). By contrast, FOXP3^+^ Tregs correlate with poor clinical outcome, and MDSC accumulation in advanced melanoma suppresses CD8^+^ T-cell proliferation (40, 44). T cells dominate both intratumoral and peritumoral inflammatory infiltrates, often within an immunosuppressive milieu enriched in Tregs and Th2-associated cytokines such as IL-4 and IL-10. Notably, imiquimod combined with photodynamic therapy shifts this landscape toward a Th1/Th17-like state characterized by IFN-γ, IL-23, IL-22, and IL-17, which is linked to inhibition of tumor growth (29, 45). Recurrent melanoma exhibits marked depletion of CD8^+^ T cells and dendritic cells at tumor sites (46). Moreover, lymphocytic infiltration is already evident in precursor lesions and persists throughout progression, while Treg density progressively increases from actinic keratosis to invasive melanoma, underscoring that lymphocyte–tumor crosstalk begins early and evolves continuously during melanomagenesis (47).

### 3.6 Neurons in melanoma

Neuroimmune signaling is increasingly recognized as an active driver of melanoma progression rather than a passive component of the tumor microenvironment. In melanoma, neurotrophic signaling, neural crest-like plasticity, and tumor-induced Schwann cell reprogramming cooperatively establish a neuro-supportive niche that promotes tumor survival, invasion, immune evasion, and metastatic dissemination. A key mediator of this process is nerve growth factor receptor (NGFR; CD271/p75NTR), which is enriched in stem-like and invasive melanoma cells (48). Mechanistically, NGFR sustains melanoma-initiating capacity by suppressing p53-dependent tumor-suppressive programs, thereby preserving cellular plasticity, clonogenicity, and resistance to stress-induced death. NGFR also facilitates immune escape: NGFR-high melanoma cells evade NK cell surveillance by downregulating NK-activating ligands and inducing stearoyl-CoA desaturase (SCD)-dependent lipid remodeling, which enhances resistance to NK cell-mediated cytotoxicity and supports metastatic fitness. Thus, NGFR links neural lineage programs to metabolic adaptation and immune resistance. Melanoma further remodels the peripheral neural niche by reprogramming adjacent Schwann cells into a repair-like state reminiscent of nerve injury. These tumor-educated Schwann cells acquire immunomodulatory and stromal-remodeling functions. Specifically, they upregulate 12/15-lipoxygenase (12/15-LOX) and COX-2, increasing immunosuppressive eicosanoid production and suppressing antitumor T cell activation, at least partly through prostaglandin E receptor 4 (EP4)-dependent signaling, thereby weakening local immune surveillance (49). In parallel, melanoma-associated Schwann cells remodel the extracellular matrix (ECM) and facilitate invasive migration. Neural signaling may also converge with β-adrenergic pathways to further amplify immunosuppression and disease progression.

## 4 Hypoxia and an acidic microenvironment in melanoma

Hypoxia and extracellular acidosis are two defining physiological features of the tumor microenvironment in melanoma. Rapid tumor expansion, together with aberrant vascular architecture, increases oxygen demand while impairing perfusion, thereby enhancing glycolytic metabolism and lactate accumulation. This hypoxic-acidic niche suppresses the proliferation and function of CD8^+^ T cells, natural killer cells, and dendritic cells, while skewing macrophages toward an M2-like immunoregulatory phenotype, collectively promoting melanoma growth and metastatic dissemination (50). In parallel, hypoxia activates HIF-dependent transcriptional programs that rewire tumor cell metabolism and stimulate angiogenesis through induction of VEGF and related mediators (51).

Hypoxia and inflammation also upregulate the ectonucleotidases CD39 and CD73, which sequentially convert extracellular ATP into adenosine. Increased CD73 expression has been associated with early resistance to PD-1 blockade in melanoma (52). Adenosine further reinforces immunosuppression through purinergic receptor signaling across multiple cell types, whereas pharmacological inhibition of this axis enhances dendritic-cell maturation, promotes cytotoxic T-cell recruitment, reduces myeloid-derived suppressor cell accumulation, and potentiates immunogenic tumor cell death (53). Acidosis further accelerates melanoma progression by promoting mesenchymal-like reprogramming, stemness-associated traits, and the production of proteolytic enzymes involved in extracellular matrix degradation (51). Notably, acidic pH has also been linked to increased PD-L1 expression in murine melanoma models and cultured melanoma cells through pH-sensitive receptor-mediated signaling, thereby facilitating immune escape (54). Buffering strategies based on alkaline compounds reduce PD-L1 abundance and suppress tumor growth *in vivo* (54). Moreover, melanoma cells adapt to hypoxia-driven acidosis by increasing the expression of intracellular buffering enzymes, among which carbonic anhydrase IX is strongly associated with metastatic aggressiveness (55). Together, hypoxia and acidosis constitute a coordinated immunometabolic program that sustains melanoma progression and therapeutic resistance.

## 5 Immunotherapy in melanoma

Clinical strategies targeting the tumor microenvironment have shown considerable promise in melanoma therapy. Among these, immune checkpoint inhibitors (ICIs) directed against PD-1, PD-L1, and CTLA-4 have reshaped the therapeutic landscape by restoring antitumor immunity and producing substantial clinical benefit in advanced melanoma (56). However, treatment responses remain heterogeneous and are influenced by multiple factors, including tumor mutational burden, PD-L1 expression, and the extent of cytotoxic T-cell infiltration within the tumor bed (57). These limitations have prompted growing interest in combinatorial approaches that target additional TME components to convert the melanoma niche from an immunosuppressive to an immune-permissive state (58).

Current TME-directed strategies focus particularly on TAMs and CAFs. TAM-targeting approaches aim to reduce infiltration of immunosuppressive M2-like macrophages, inhibit their protumor functions, and enhance M1-like antitumor activity. CSF1R inhibition has shown efficacy in reducing macrophage abundance in multiple tumor models, although systemic administration of pexidartinib may also deplete tissue-resident macrophages in normal organs. To mitigate this toxicity, localized hydrogel-based delivery of pexidartinib nanoparticles has been developed and shown to reduce adverse effects (59). Additional macrophage-directed strategies include blockade of the CCL2/CCR2 axis to prevent monocyte recruitment, as well as macrophage reprogramming using CD40 agonists and PI3K pathway inhibitors (8, 60). Notably, the PI3Kγ inhibitor IPI-549 reprogrammes macrophages toward a pro-inflammatory phenotype and, in preclinical models resistant to ICIs, restores sensitivity to checkpoint blockade and delays tumor progression (61). This approach is currently under evaluation in a Phase I trial (NCT02637531). Moreover, inhibition of the CD47–SIRPα axis enhances macrophage-mediated phagocytosis by disrupting the tumor-derived “don’t eat me” signal (62).

Therapies targeting CAFs aim either to deplete fibroblast populations or to restrain their tumor-promoting functions. Fibroblast activation protein targeting reduces FAP^+^ CAF abundance, whereas talabostat has shown fibroblast-reducing effects in preclinical models (63). By contrast, imatinib has demonstrated limited efficacy in blocking CAF differentiation, suggesting that more selective PDGFRβ-directed or combinatorial strategies may be required (64). In parallel, disruption of CAF-derived cytokines such as IL-6 and CXCL12 may further improve tumor control. Other interventions target non-cellular TME components, including extracellular matrix remodelling enzymes and ECM-regulated signalling pathways (65). For example, the MMP2/MMP9 inhibitor S-3304 showed preliminary activity in a Phase I study of advanced solid tumors, although its therapeutic value requires further validation (66). Finally, therapies targeting hypoxia include hypoxia-activated prodrugs, HIF pathway modulation, and inhibition of carbonic anhydrase IX. These agents selectively eliminate malignant cells within oxygen-deprived tumor regions while sparing normal tissues (67). Collectively, these approaches highlight the expanding therapeutic potential of TME-directed interventions in melanoma.

## 6 Conclusion

The immune landscape of the melanoma microenvironment represents a highly organized and dynamically evolving ecosystem, wherein complex crosstalk among tumor-associated macrophages, cancer-associated fibroblasts, regulatory T cells, endothelial cells, neurons, and extracellular matrix components collectively establishes a profoundly immunosuppressive and tumor-permissive niche. This integrated network not only drives melanoma proliferation, invasion, and metastatic dissemination but also actively undermines antitumor immunity by suppressing cytotoxic lymphocyte function, recruiting regulatory immune populations, and fostering metabolic stress conditions such as hypoxia and acidosis. Mechanistically, key pathways, including lipid metabolic reprogramming in macrophages, CAF-derived chemokine signaling (CXCL12/CCL17), adenosine-generating CD39/CD73 axis, and NGFR-mediated neuroimmune adaptation, converge to reinforce immune evasion and therapeutic resistance. These insights fundamentally challenge a tumor-centric view and underscore that effective melanoma control requires targeting the broader microenvironment as an interconnected system rather than isolated cellular components.

Given the remarkable heterogeneity and adaptability of the melanoma immune microenvironment, future therapeutic strategies must move beyond single-agent immune checkpoint blockade toward rationally designed, multimodal interventions that remodel the entire stromal-immune landscape. Promising directions include reprogramming tumor-associated macrophages toward a pro-inflammatory M1-like state using PI3Kγ inhibitors or CD40 agonists, disrupting CAF-mediated immunosuppression via IL-6 or CXCL12 blockade, alleviating hypoxic stress through carbonic anhydrase IX inhibition or hypoxia-activated prodrugs, and buffering acidosis to restore T-cell function. Ultimately, advancing spatial transcriptomics and patient-specific functional profiling will be essential to decode interpatient heterogeneity and guide personalized combinatorial regimens capable of converting the “cold” immunosuppressive melanoma niche into a “hot” immune-responsive state, thereby achieving more durable and effective clinical outcomes.
